# The Underreported Postoperative Suffering after Thyroid Surgery: Dysphagia, Dysphonia, and Neck Pain—A Cross-Sectional Study

**DOI:** 10.1155/2023/1312980

**Published:** 2023-08-07

**Authors:** Hunduma Jisha Chawaka, Zenebe Bekele Teshome

**Affiliations:** Ambo University, Anesthesiology, Ambo, Ethiopia

## Abstract

**Background and Aims:**

Postoperative voice change, difficulty of swallowing, throat pain, and neck pain are the most commonly complaint after thyroid surgery. However, little emphasis is given to the problem, especially a place where the surgical and anesthesia services' unmet need is highly observed, i.e., the problem gets little attention especially in the countries where the gaps of surgery and anesthesia services are observed. Hence, this study aims to determine the magnitude and associated factors of voice change and related complaints after thyroid surgery.

**Methods:**

A cross-sectional study was conducted on 151 patients who had had thyroid surgery from June 1 to December 30, 2021. Data were retrieved during the postoperative period after the patient regains consciousness.

**Result:**

Out of 151 participants, 98 (64.9%) patients complained of either voice change or difficulty of swallowing and neck pain after thyroid surgery within 24 hours. Majority (58.3%) of the participants aged more than 30 years with a mean age of 33.7 ± 8.3 years and females 102 (67.5%). Neck pain is the most (52.3%) complained suffering after thyroid surgery, followed by voice change 38.4% and difficulty in swallowing 37.7%. Difficulty in swallowing after thyroid surgery significantly associated with a patient who frequently experience intraoperative hypotension (AOR = 23.24, 95% CI 4.6–116.7, and *p* = 0.01), type of surgical procedure (total thyroidectomy) (AOR = 8.62, 95% CI 1.21–61.50, and *p* = 0.03), and larger ETT size (AOR = 4.92, 95% CI 1.34–18.01, and *p* = 0.02). Postoperative voice change is associated with larger endotracheal tube (AOR = 15.47, 95% CI 3.4–69.5, and *p* ≤ 0.001), surgery lasting more than 2 hours (AOR = 7.34, 95% CI 1.5–35.1, and *p* = 0.01), and intraoperative hypotension (AOR = 23.24, 95% CI 4.6–116.7, and *p* ≤ 0.001).

**Conclusion:**

The complaint of postthyroidectomy neck pain and throat discomfort is higher than 64.9%. Intraoperative hypotension, blood loss, higher ETT size utilization, and duration of surgical procedure are the identified possible risk factors and have to be minimized as much as possible. Patient reassurance has to be considered during the postoperative time.

## 1. Background

Though low incidence of permanent or temporary recurrent laryngeal nerve (RLN) injury (0–15.4%) and superior laryngeal nerve injury (0–4.6%) is reported, voice change and difficulty in swallowing after total thyroidectomy are increasingly prevalent with studies reporting rates up to 87% [1–6]. However, little emphasis is given to this problem. The difficulty in swallowing and voice change after thyroidectomy can significantly interfere with one's own quality of life, affecting social interactions, medication compliance, and nutritional intake [7].

An undetermined broad range of symptoms is encountered with varying severity levels of clinical scenarios after thyroid surgery is a common problem [8].

Airway-related complaints (voice change, swallowing difficulty, and neck pain) are a common distressing sequel after thyroid surgery that significantly contributes to postoperative patient dissatisfaction. Though many patients recover within a few days, it can interfere with the patient's activity after leaving the hospital. A postoperative respiratory compromise could significantly impact the well-being of patients after surgical procedures especially under general anesthesia. Anesthesia complication is the patient's physiologic derangement or any unwanted effect related to anesthesia management, of which airway-related complain is among the most encountered problems after thyroidectomy [9–11].

A plenty of research had done worldwide overwhelmingly regarding the sore throat, whereas very few studies about post-thyroidectomy neck pain, voice change, and swallowing-related complaints have been addressed, if any, in sub-Saharan countries. However, it was well investigated in developed countries and showed the incidence of postoperative voice changes. Some researchers found that it increases with being female and age older (>50 years) [12].

Another study described that the postthyroidectomy voice change and throat complaints are associated with larger ETT tubes, age older than 30 years, extensive surgical approach, and longer (>1 hour) duration of surgery. The study conducted on 140 thyroid surgeries in Pakistan, at a university hospital, reported that larger ETT, prolonged surgery, and surgical procedure types were well documented that it is associated with post-thyroidectomy complaints [13].

Factors that contribute to the development of postoperative airway-related complaints include the technical difficulty of intubation [14], high endotracheal tube cuff pressures [15], longer duration of surgical procedures [16], direct injury to mucosa with laryngoscopy, or pharyngeal airway and larger endotracheal tube use [16–19].

The practices of airway management methods and surgical approaches for a particular procedure have often shown highly variable from institution to institution and person to person. In addition to this, there is limited information about the incidence and contributing factors of post-thyroidectomy airway-related complaints in sub-Saharan countries like Ethiopia, where insufficient resources and qualified manpowers are the main problems.

Furthermore, other contributing factors such as extubation time, blood loss, intraoperative hypotension, ASA status of the patient, cough on ETT, and others had not been taken into consideration in the so-far research. But still, I consider there is an increased incidence of postoperative airway-related discomfort in those patients. Our study focused on further determining the presence of association and describing the other variables.

Therefore, this study was conducted as a preliminary step to determine the magnitude and determinants of under-recognized postoperative neck pain, swallowing difficulty, and voice changes after thyroid surgery at Ethiopian teaching hospitals.

## 2. Methods

### 2.1. Study Setting and Design

This institutional-basedprospective cohort study was conducted at the University Referral and Teaching Hospital (URH), located 114 km away from Addis Ababa to West of Ethiopia, from May 01 to December 30, 2021. The hospital provides a range of health care services for >5 million people, including major surgical treatments: orthopedic surgery, general surgery, gynecologic, and obstetric surgery. On average about six major elective surgical procedures performed each day, of which one third is thyroid surgery.

### 2.2. Source Population

Source population includes all patients who undergo surgery under general anesthesia during the study period.

### 2.3. Study Population

Study population includes patients who undergo thyroid surgery who met inclusion criteria during the study period.

### 2.4. Inclusion and Exclusion Criteria

#### 2.4.1. Inclusion Criteria

All patients whose age is 18 years and or above who undergo thyroid surgery were included in the study.

#### 2.4.2. Exclusion Criteria

ASA-3 or above, recent or ongoing URTI, patient with history of recent nasogastric tube use, double lumen ETT, and preexisting voice change and or difficulty of swallowing were excluded from the study.

## 3. Sample Size and Procedure

**Figure 1:**
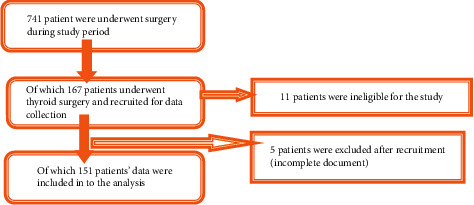
Diagram illustrating sampling process.

We have 4 major OR theatre (table) for both emergency and elective surgical procedures. On average about 6 elective surgery were performed each days, and the preliminary survey data showed 313 overall surgical procedure performed during the working days over the last three months, before the study period. For this reason, we included all thyroid surgery patients **(**Figure 1).

## 4. Study Variable

### 4.1. Dependent

The dependent variable includes presence of voice change, neck pain, and difficulty of swallowing after thyroid surgery (yes/no).

### 4.2. Independent

The independent variable includes age, sex, diagnosis, duration of anesthesia, duration of surgery/ETT, ETT size, estimated blood loss in milliliter, frequency of hypotension episodes, extubation mode (deep/awake), cough while ETT inside, types of surgical procedure, and anesthetist's experience.

## 5. Data Collection and Analysis Process

Data were collected through patient interviews and card review using a semistructured questionnaire during postoperative time, within 24 hours, after the patient fully recovered from anesthesia and felt comfortable to answer the interviewer's question. Demographic data: age, sex, weight, height, American Society of Anesthesiologists (ASA)'s physical status, surgical procedure type, duration of intubation, size of the cuffed endotracheal tube (ETT) utilized, mode of extubation, intraoperative recorded hypotension, estimated intraoperative blood loss (EBL), and anesthetists year of experience were recorded on a standard form (see Annex 1). The interviewer asks a direct question about voice change and or swallowing-related symptoms, including “hard to chew,” “hard to swallow,” “lump in the throat,” “trouble swallowing,” and any voice change or hoarseness after surgery. The data were collected from all patients who underwent surgery to assess the airway-related and postoperative discomfort; later on, the thyroid surgery was identified to be analyzed separately and the manuscript was rewritten for publication.

A pretest was done on eight patients before actual data collection, and necessary corrections and modifications were made to the questionnaires.

Each questionnaire was checked for completeness and entered into SPSS.V.20 statistical software for analysis. A logistic regression analysis was used to determine the association of various factors with the outcome variable. Each descriptive variable with an outcome variable was assessed for its association, and those with *p* value <0.05 were reported with 95% CI as a statistically significant variable.

## 6. Result

Seven hundred forty-one surgeries were performed during the study period, of which 167 patients underwent thyroid surgery. Of these, 11 patients were ineligible for the study and 5 patients were excluded after recruitment (incomplete document). Lastly, 151 patients' data were included in this study (with a mean age of 33.7 ± 8.27 years). Majority of the participants were females (68.9%), ASA-I (American Society of Anesthesiologists) (62.3%), and 18–24 BMI (84.8%).

Hyperthyroidism in 76 (50.3%), goiter (Graves' disease) in 42 (27.8%), suspicion of malignancy in 20 (13.3%), and hyperparathyroidism in 13 (8.6%) were among the mentioned diagnosis or indications before surgery.

The most frequently performed surgical procedures include total thyroidectomy in 68 (45%), near-total or semithyroidectomy in 79 (52.4%), and isthmectomy in 4 (2.6%). The surgery was performed in a supine position with a duration range of 1½ to 3½ hours, and 30.5% of the participants had recorded intraoperative hypotension episodes of which 25.2% were more than two times. All patients were anesthetized using standard intravenous anesthetics and intubated using a conventional laryngoscope (blade-3 mackintosh) and ETT size of 6.5 or 7.0 mm (Table 1).

### 6.1. Outcome and Possible Risk Factors

**Figure 2:**
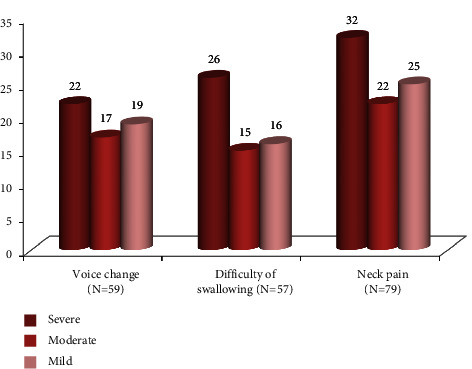
Percentage distribution of postoperative complain severity level after thyroid surgery.

An overall 98 (64.9%) patients complained of either voice change or difficulty in swallowing and neck pain within 24 hours after thyroid surgery. Postoperative neck pain is the most (52.3%) complained suffering after thyroid surgery and followed by voice change 38.4% and difficulty in swallowing 37.7%. The majority of the complaint noticed immediately (within one hour), as soon as they got their consciousness and it was mild in most of them; however, voice change is getting severe over times (Figure 2).

Frequent episodes of hypotension (AOR = 23.24, 95% CI 4.6–116.7), type of surgical procedure (total thyroidectomy), larger ETT size, and laryngoscopy by less experienced anesthetists have found to be statistically associated with the incidence of postoperative difficulty in swallowing.

In addition to the abovementioned factors, postoperative voice change is associated with those patients whose surgical procedure lasts more than 2 hours, whereas neck pain after thyroidectomy is significantly associated with the duration of surgical procedures (Table 2).

## 7. Discussion

Although post-thyroidectomy laryngeal nerve injury and cord paralysis had been extensively studied, airway-related complaints have not been given attention and little is known, if any. Sore throat, voice change, difficulty of swallowing, neck pain, and difficulty to cough are among the commonest problems after endotracheal tube extubation (ETT) of thyroidectomy patients [20–26].

A worldwide incidence of post-thyroidectomy airway-related complaints is overwhelmingly varied: dysphagia and dysphonia have been documented as high as 93.3% and 43.3%, respectively [27]. The problem is also reported as low as 174 (18.8%) by Sahli et al., 2020, from 924 patient [28].

We found 61.6% of patients complained of either voice change or difficulty swallowing within 24 hours after thyroid surgery; of which, 38.4% had voice change and 37.7% had difficulty swallowing. In addition to these, majority (52.3%) of the patient suffers from neck pain.

In congruent to this finding, there was a report by Holler and Anderson, 2014, from St. Michael's Hospital, Canada, revealed that voice and swallowing complaints after thyroid surgery were 32.2% and 42.7%, respectively [29]. Another German university hospital by Hillenbrand et al., 2013, showed a comparable incidence of 50.2% (110/219) of dysphagia in the immediate postoperative time [30].

However, fewer complaints of voice or swallowing change after thyroidectomy were reported by Sahli et al., 2020, from 18.8% (174/924) of the overall compliant, 148 (16.0%) voice alteration and 51 (5.5%) swallowing [28]. Another study conducted by Scerrino et al., 2013, in Italy, found that 20% of the complaints suffer voice change and 30% had difficulty in swallowing after thyroidectomy [31].

A much higher report was also documented by Souza BBA et al., 2006, postoperative dysphagia (93.3%) and dysphonia (43.3%) [27]. This discrepancy might be from the difference in technical surgical procedure and assessment time.

Extensive studies have been conducted on laryngeal nerve injury associated with voice change, swallowing difficulties, and other possible risk factors. However, little, if any, was addressed regarding the nonlaryngeal nerve injury complaints after thyroid, especially in developing countries. Total thyroidectomy, older age, and being female were identified as risk factors of post-thyroidectomy low-pitch voice changes [12].

This paper founds that postoperative dysphagia is significantly associated with patients who have frequent episodes of hypotension, type of surgical procedure (total/near-total thyroidectomy), larger ETT size, and laryngoscopy by the less experienced anesthetist. This can be explained by hypotension exaggerating the compressive effect of laryngeal mucosa by ETT cuff that might result in ischemia. Total thyroidectomy and laryngoscopy by beginners might be associated with direct mucosal injury and swelling from inflammation which results in dysphagia. A similar finding was reported by Kadri IA et al., 2009, from Pakistan [13]. With a similar reasoning, postoperative voice change is found to be associated with larger endotracheal tube utilization, surgery lasting more than 2 hours, and frequent or persistent hypotension. Intraoperative hypotension and larger endotracheal tube were found to be exclusive risk factors for the occurrence of post-thyroidectomy voice change and difficulty in swallowing. In addition to this, postoperative neck pain is the most commonly complained sufferings after thyroid surgery which was found statistically associated with longer-lasting surgical duration. This might be because of cervical nerve root compression in a usual positioning, pillow under the scapula.

It has been documented by many researchers that intubation duration has a significant association with postoperative throat pain and voice change occurrence after extubation [16, 32–34].

## 8. Conclusion and Recommendation

It would be crucial that the clinicians understand the consequences of thyroidectomy from the patient's perspective other than nerve injury to better care for these patients. The complaint of neck pain and throat discomfort after thyroidectomy is higher than 64.9%. Intraoperative hypotension, blood loss, higher ETT size utilization, and duration of surgery are the identified possible risk factors and have to be minimized as much as possible. Besides this, patient reassurance and increased awareness of the sequel of thyroidectomy would help in minimizing the anxiety, frustration, and insecurity surrounding postoperative dysphagia, voice alteration, and neck pain.

## Figures and Tables

**Table 1:** Percentage and frequency distribution of demographic, anesthetic, and surgery-related variables of postoperative airway discomfort after thyroidectomy.

Variables	Variable category	Frequency (*N*)	Percent (%)
Gender	Female	102	67.5
	Male	49	32.5

Age	>30 years	88	58.3
	<30 years	63	41.7

BMI	≥30	6	4.0
	24–30	17	11.3
	18–24	128	84.8

ASA status	II	57	37.7
	I	94	62.3

ETT size ID	7.0 or 7.5	55	36.4
	6.0 or 6.5	96	63.6

Duration of surgery	>3 hours	23	15.2
	2-3 hours	41	27.2
	1-2 hours	87	57.6

Extubation mode	Deep	108	71.5
	Awake	43	28.5

Types of procedure	Total	35	23.2
	Subtotal	58	38.4
	Lobectomy	58	38.4

Intraoperative hypotension (<90/60 mmHg)	Yes	70	46.4
	No	81	53.6

Frequency of hypotension	More than 2 times	53	35.1
	Less than 2 times	17	11.3
	Not at all	81	53.6

Is EBL >15% of EBVt	Yes	64	42.4
	No	87	57.6

Anesthetists' experience	Less than 2 years	52	34.4
	More than 2 years	99	65.6

Throat pain	Yes	74	49
	No	77	51

**Table 2:** Multivariate output of factors associated with postoperative complain after thyroid surgery.

Postoperative complain	Variables (risk factors)	COR	AOR	95% CI	*P* value
Voice change	Large ETT size	17.29	15.47	3.4–69.5	0.001
	Duration of surgery lasting 2 hours or more	7.22	7.34	1.5–35.1	0.01
	Frequent intraoperative hypotension	4.92	23.24	4.6–116.7	0.001
	Postoperative throat pain	0.36	0.41	0.07–2.58	0.34

Swallowing difficulty	Large ETT size	4.65	4.92	1.34–18.01	0.02
	Types of procedure = total/near total	2.09	8.62	1.21–61.50	0.03
	Frequent intraoperative hypotension	2.42	6.96	1.77–27.39	0.01
	Less experienced anesthetist (2 year)	3.33	5.40	1.16–25.06	0.03

Neck pain	Large ETT size	0.06	0.76	0.43–1.35	0.36
	Duration of surgery lasting 2 hours or more	9.90	10.26	2.15–48.88	0.001
	Types of procedure	0.53	0.35	0.1–1.14	0.08
	Anesthetists' experiences	1.90	1.17	0.41–3.38	0.77

*Note.* ETT: endotracheal tube; EBL: estimated blood loss; EBVt: estimated total blood volume.

## Data Availability

The data used to support the findings of this study are available from the corresponding author upon request.

## Abbreviations

AOR: Adjusted odd ratio
ASA: American Society of Anesthesiologist physical status
AURH: Ambo University Referral Hospital
COR: Crudes odd ratio
EBL: Estimated blood loss
EBV: Estimated blood volume
ETT: Endotracheal tube
GA: General anesthesia
HPT: Hypotension
PACU: Postanesthesia care unit
POST: Postoperative sore throat
VAS: Visual analog scale.

## Supplementary Materials

Supplementary Materials There is a separately submitted questionnaire (annexes), which contain in detail the collected data.
