# Supplementary material for: The Underreported Postoperative Suffering after Thyroid Surgery: Dysphagia, Dysphonia, and Neck Pain—A Cross-Sectional Study
Source: Anesthesiol Res Pract. 2023 Aug 7;2023:1312980. doi: 10.1155/2023/1312980 (PMC10425250; doi:10.1155/2023/1312980)
Supplement: Supplementary Materials — There is a separately submitted questionnaire (annexes), which contain in detail the collected data. [file 1312980.f1.doc]

# Annex 1; Questionnaire:

**Consent**

**Greetings;** Hello! My name is ---------------------------------------------- briefing objectives of study and process……….

If you agree, I will start my questions by asking general identification points.

“May I continue?” Yes / No

If yes, continue interviewing

If no, thanks and stop interviewing

- Interviewer signature _____________________________ Date____________________
- Questionnaire code ________________ Medical Identification Card ____________________
- Time of start of interview _____________ Time of end of interviewed______________

**I. Patient identification (character)**

02. What is your age (year)? ____________ **Sex**: Male____Female____

03. Do you know/ heard what anesthesia is?_____Y/N______

04. Have had ever surgery before Yes/NO? Site of surgery _____________________

Type of surgical procedure___________________________

**Part I. Questionnaire on Socio-Demographic characteristics**

**101**. What is the highest level of schooling you have ever attended?

1. Illiterate 2.elementary school (1-8) 3. Secondary high school (9-12) 4. College diploma 5. Degree and above

**102**. What is your occupation?

1. House wife 2. Farmer 3. Civil Servant 4. Merchant 5 Other

**103.** What is your marital status?

1. Never married 2. Married 3. Divorced 4. Widowed 5. Separated

**104**. Which ethnic group do you belong to?

1. Oromo 2. Amhara 3. Gurage 4. Somali 5. Others

**105.** What is your religious affiliation?

1. Protestant Christian 2. Orthodox Christian 3. Catholic Christian 4. Muslim 5. Others (Specify)______________

**106**. Do you have any complain after surgery or anesthesia? _________Y/N___________

**107**. (If yes), indicate one or more that you have suffered;

1. Throat discomfort 2.Wound site pain 3. Nausea vomiting 4. Thermal discomfort 5.Dry mouth 6.Head ache

**108.** Indicate; which complain most threaten your comfort during your last surgery?

1. Throat discomfort 2.Wound site pain 3. Nausea vomiting 4. Thermal discomfort 5.Dry mouth 6.Head ache

**109**. Do you have pain to surgical site? If yes, indicate severity level…..NRS

0. No 2. Minor 3. Moderate 4. Severe 5. Worst

**110.** Do you have pain in your **throat**? _____Y/N ____ If yes, indicate severity level;

0. No 2. Minor 3. Moderate 4. Severe 5. Worst

**111.** When does this throat discomfort feeling started /occurred _____________(hour)

**112**. Do you have any voice change? _____Y/N ____ If yes, indicate severity level;

0. No 2. Minor 3. Moderate 4. Severe 5. Worst

**113.** When does this voice change occurred feeling started /occurred ____________(hour)

114. Do you have neck pain? ______Y/N___ If yes, indicate severity level;

0. No 2. Minor 3. Moderate 4. Severe 5. Worst

III. Information to be collected from patient card about anesthesia and surgical

| **SN** | **Variable** | **Yes** | **No** |
| --- | --- | --- | --- |
|  | BMI |  |  |
|  | Physical ASA status |  |  |
|  | Was analgesic given preop/ intraoperative |  |  |
|  | Post-operative pain controlled (documented) |  |  |
|  | Postoperative pain level (documented) |  |  |
|  | Induction anesthetic agent used |  |  |
|  | Laryngoscopic view grade |  |  |
|  | Number of intubation attempted |  |  |
|  | ETT size (internal diameter; ID in mm) |  |  |
|  | ETT depth (tip of tube placement from angle of mouth; in cm) |  |  |
|  | Duration of intubation / duration of surgery |  |  |
|  | Extubation mode (awake/deep) |  |  |
|  | Does patient coughed while ETT insitu |  |  |
|  | Blood were observed on ETT / laryngoscope tips |  |  |
|  | Any documented BP drops (<90/60) persist for more than 3mint |  |  |
|  | Estimated blood loss > 15% of EBVt? |  |  |
|  | Anesthetists’ educational status –senior /junior |  |  |
|  | Types of surgical procedure (thyroidectomy) |  | |
|  | Surgery performed by –senior /junior |  | |
